# Renal Replacement Therapy in Patients With Acute Decompensated Pulmonary Hypertension Admitted to the Intensive Care Unit

**DOI:** 10.7759/cureus.28792

**Published:** 2022-09-05

**Authors:** Marcos Garcia, Rogerio Souza, Pedro Caruso

**Affiliations:** 1 Pulmonary Division, Heart Institute, Faculty of Medicine, University of São Paulo, São Paulo, BRA; 2 Intensive Care Unit, A.C. Camargo Cancer Center, São Paulo, BRA

**Keywords:** renal replacement therapy (rrt), chronic thromboembolic pulmonary hypertension, cteph, group i pulmonary hypertension, – pulmonary hypertension

## Abstract

Background: Pulmonary arterial hypertension and chronic thromboembolic pulmonary hypertension (PH) are characterized hemodynamically by pre-capillary PH. Acute worsening of systemic congestion and/or reduced right ventricular flow output in patients with pre-capillary PH characterizes an episode of acute decompensated PH. Acute kidney injury (AKI) is a common complication in this population and those patients frequently use renal replacement therapy (RRT). Predictors and timing for RRT in acute decompensated PH are unknown and mortality of patients who require this therapy is high. We hypothesize that AKI and hypervolemia are associated with use of RRT during episodes of acute decompensated PH in patients with pre-capillary PH requiring intensive care unit (ICU) admission.

Aim: Explore variables associated with RRT use, develop a decision tree model to predict use of RRT in acute decompensated PH and analyze ICU, in-hospital and 90-days mortality in this population.

Materials and methods: Multicenter retrospective cohort study including patients with pulmonary arterial hypertension and chronic thromboembolic PH with unplanned admission in the ICU for acute decompensated PH. Acute decompensated PH was defined by acute right ventricular failure leading to low cardiac output and elevated right ventricle filling pressures. We employed two multivariable logistic regression models using directed acyclic graphs to identify confounders. Unadjusted and adjusted odds ratios and 95% confidence intervals were used to measure the association between variables and RRT use.

Results: Some 73 patients were included, 16.4% (n=12) of patients required RRT during ICU stay. In the univariate analysis, right atrial pressure (RAP) on last right heart catheterization, and creatinine upon ICU admission were associated with use of RRT and were included in the multivariable model and in the decision tree model. The decision tree model based on RAP and creatinine showed sensitivity of 58.3% and specificity of 100% with area under the receiver operating characteristic curve of 0.81 for predicting RRT use in the ICU. In-hospital mortality and 90-days mortality of patients who used RRT were higher than in patients that did not use RRT (75.0% vs. 34.4%, p < 0.01 and 83.3% vs. 42.6%, p = 0.01, respectively).

Conclusion: The decision tree model based on creatinine upon admission and RAP, which is a surrogate of hypervolemia, can identify patients at risk for RRT. Increased ICU, in-hospital, and 90-days mortality were observed in patients with acute decompensated PH who used RRT in the ICU.

## Introduction

Pulmonary arterial hypertension (group 1) and chronic thromboembolic pulmonary hypertension (PH) (group 4) are characterized hemodynamically by pre-capillary PH [1-2]. Conversely, PH caused by left heart disease (group 2), is characterized by isolated post-capillary PH or, rarely, combined pre- and post-capillary PH [1]. Lung disease related PH (group 3) is a complex and heterogenous group, incompletely understood and with unclear treatment strategy, which depends on the causative lung disease [3].

Acute worsening of systemic congestion and/or reduced right ventricular flow output in patients with pre-capillary PH characterizes an episode of acute decompensated PH [4-5]. Acute kidney injury (AKI) is a common complication in this population and those patients frequently use renal replacement therapy (RRT) [6].

Systemic venous congestion, decreased cardiac output, activation of the neurohormonal axis, and renal retention of sodium and water are the implicated mechanisms that can lead to AKI and hypervolemia during acute decompensated pulmonary hypertension (ADPH) episodes [7-8]. Chronic systemic venous congestion can be clinically represented by increased right atrial pressure (RAP). In patients with pulmonary arterial hypertension (PAH), both decreased cardiac index and increased RAP are independent determinants of reduced estimated glomerular filtration rate over the course of the disease [9].

In the setting of ADPH, acute worsening hypervolemia and AKI can lead to refractory congestive right heart failure [10]. RRT is usually initiated when AKI, hypervolemia, or both, are refractory to supportive therapy. Predictors for need of RRT and timing for RRT in ADPH are unknown and mortality of patients who require this therapy is high [6-7, 11-14]. 

There are potential risks in administrating RRT in this population including hemodynamic instability, bleeding, and complications related to venous access and dialysis circuit [6]. We hypothesized that hypervolemia and AKI are associated with use of RRT during episode of ADPH in patients with pre-capillary PH requiring ICU admission.

## Materials and methods

To study the hypothesis that hypervolemia and AKI are associated with use of RRT therapy during episode of ADPH in patients with chronic pre-capillary PH, and to explore variables potentially associated with RRT use, we performed a multicenter retrospective cohort study including patients with PAH (group 1) and non-surgical chronic thromboembolic pulmonary hypertension (CTEPH) (group 4), with unplanned admission to ICU. Secondarily, we developed a decision tree model to predict use of RRT and we also described ICU, in-hospital, and 90-days mortality in this population. 

Patients were admitted to ICU between 2014 and 2019, involving three ICUs of a large medical complex in Sao Paulo, Brazil. Ethics committee approved the study and waived the need for informed consent. This was a retrospective study, and there was no consequence on patient management.

Chronic pre-capillary PH corresponded to PAH (group 1) and non-surgical CTEPH (group 4), as previously described [14]. In order to study ADPH only in patients with pre-capillary PH and keep a homogeneous sample to allow better interpretation of results, we decided to not include other groups of PH in this study. 

Acute kidney injury was defined by RIFLE criteria. ADPH was defined by acute right ventricular failure leading to low cardiac output and elevated right ventricle filling pressures [5]. We collected clinical, functional, hemodynamic, and laboratory data before ICU admission (from last six months prior to ICU admission), upon ICU admission, and during ICU stay. 

Categorical and continuous data are presented as frequencies (percentages) and median (25%-75% interquartile range). Categorical variables were compared with chi-square test or Fisher’s exact test. Continuous variables were compared with Mann-Whitney test. To test the hypothesis that AKI and hypervolemia are associated with RRT use, we employed two multivariate logistic regression models using directed acyclic graphs to identify confounders [15]. Unadjusted and adjusted odds ratios and 95% confidence intervals were used to measure the association between each variable and RRT use.

Next, significant variables (p < 0.05) were entered into a machine learning derived decision tree to predict RRT use. The decision tree method used the k-fold cross method for internal validation and Bonferroni correction to adjust for multiple testing. Discrimination and calibration were estimated using area under the receiver operating characteristic curve (AUC-ROC) and R2, intercept, and slope. 

The 90-days mortality curves were generated using the Kaplan-Meier method and compared using the log-rank test. Statistical analyses were performed using SPSS software (Version 23.0, IBM, Armonk, NY). P-values ≤ 0.05 were considered significant.

## Results

A total of 73 patients with ADPH were included and 12 patients (16.4%) required RRT during ICU stay. No patients were using RRT before ICU admission. Last ambulatory RAP, a surrogate of hypervolemia, and ICU admission creatinine were higher in the RRT group compared to no RRT group. Most patients who required RRT had CTEPH n=8/12 (66.7%). Baseline and ICU admission data are depicted in Table 1.

**Table 1:** Baseline and ICU admission data. Categorical and continuous data are presented as frequencies (percentages) and median (25%-75% interquartile range). PH, pulmonary hypertension; PAH, pulmonary arterial hypertension; CTPEH, chronic thromboembolic pulmonary hypertension; ERS risk assessment, European Society of Cardiology/European Respiratory Society pulmonary hypertension risk assessment; BNP, brain natriuretic peptide in pg/mL; PAP, pulmonary artery pressure in mmHg; PVR, pulmonary vascular resistance in woods; PAWP, pulmonary artery wedge pressure in mmHg; RAP, right atrium pressure in mmHg; ICU, intensive care unit; SOFA, sequential organ failure assessment; IV, intravenous; RRT, renal replacement therapy ^†^Vasopressors defined as any use of norepinephrine, vasopressin, or epinephrine.

Variables	RRT Group (n=12)	No RRT (n=61)	p
Age (years)	51 (43-61)	47 (34-58)	0.42
Female	6 (50.0%)	49 (80.3%)	0.05
PH group			0.02
PAH	4 (33.3%)	43 (70.5%)	
CTEPH	8 (66.7%)	18 (29.5%)	
ERS risk assessment			0.09
Low	0 (0%)	7 (11.5%)	
Intermediate	4 (33.3%)	38 (62.3%)	
High	8 (66.7%)	16 (26.2%)	
Creatinine (mg/dL)	1.41 (1.17-2.14)	1.20 (0.90-1.71)	0.13
BNP (pg/mlL	460 (231-478)	389 (193-624)	0.86
Hemodynamics			
Median PAP (mmHg)	60 (45-66)	56 (50-66)	0.95
PVR (Woods)	11.9 (7.7-13.6)	12.8 (9.3-19.3)	0.13
PAWP (mmHg)	15 (13-17)	11 (8-15)	0.06
RAP (mmHg)	22 (18-25)	13 (8-17)	<0.01
Cardiac output (L/min)	4.1 (3.6-4.7)	3.6 (2.8-4.6)	0.29
	ICU admission data		
PH decompensation reason, n (%)			0.94
Unknown	3 (25%)	12 (19.7%)	
Infection	6 (50%)	29 (47.5%)	
Hypervolemia	2 (16.7%)	12 (19.7%)	
Arrhythmia	1 (8.3%)	5 (8.2%)	
Pregnancy	0 (0%)	3 (4.9%)	
SOFA	7 (6-10)	6 (4-8)	0.10
Creatinine (mg/dl)	3.16 (2.12-3.9)	1.26 (0.89-2.16)	<0.01
BNP (pg/mL) (n=62)	529 (393-1340)	690 (354-1143)	0.86
Sodium (mEq/L)	136 (133-138)	137 (133-139)	0.69
Arterial lactate (mg/dL)	18 (11-23)	18 (14-23)	0.68
IV furosemide use	12 (100%)	59 (96.7%)	0.34
Dobutamine use	12 (100%)	50 (82.0%)	0.12
Vasopressors use ^†^	21 (70.0%)	12 (27.9%)	0.09

In the RRT group, the median central venous pressure at admission was 18 mmHg (17-26 mmHg), all patients in the RRT group were receiving IV furosemide upon ICU admission. Median net fluid balance of first 24 h was -615 mL (-1091 to -91) in RRT group. The main reasons for RRT initiation were low urine output and need for negative fluid balance (n=9/12), refractory metabolic acidosis (n=2/12), and refractory hyperkalemia (n=1/12).

The median number of days between ICU admission and RRT initiation was 2 (0-7) and the median duration of RRT was 5 days (4-7). Seven patients were under anticoagulation with heparin (n=4) or citrate (n=3). The most common RRT method was continuous venovenous hemodiafiltration (n=9/12), with a median fluid removal rate of 100 mL/h (33-120) in 24 h. In three patients, the RRT method was sustained low efficiency dialysis, with a median fluid removal rate of 250 mL/h (230-267); median duration of RRT session was 7 h (6-8) daily. Adverse events were reported in eight patients: transient hypotension (n=4), clotting of the system (n=2), malfunctioning line (n=1), and bleeding from line insertion (n=1).

Increased creatinine was associated with RRT requirement [unadjusted odds ratio 2.87 (1.49-5.52; 95% confidence interval, CI)]. Adjusting for gender and PH group, increased creatinine was associated with RRT requirement [adjusted odds ratio, OR 2.70 (1.35-5.40; 95% CI)]. Increased RAP was associated with RRT requirement [unadjusted OR 1.12 (1.02-1.24; 95% CI)]. Adjusting for gender and PH group, increased RAP was not associated with RRT requirement [adjusted OR 1.08 (0.97-1.20; 95% CI)].

**Figure 1:**
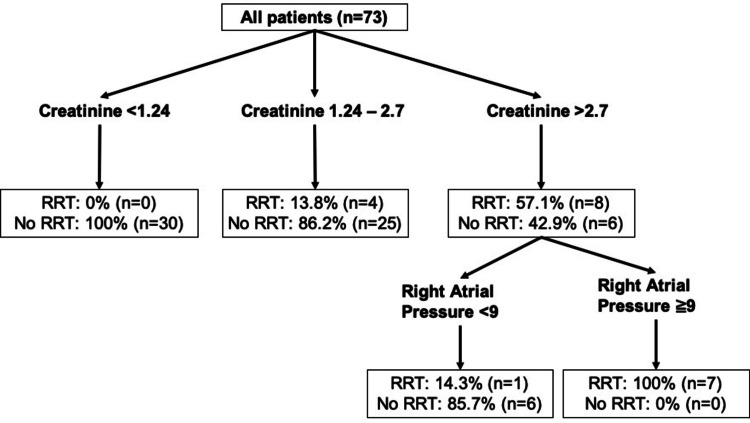
Decision tree model of recursive partitioning analysis for predicting RRT based on ICU admission creatinine and right atrial pressure on last right heart catheterization. RRT, renal replacement therapy; ICU, intensive care unit

Creatinine and RAP were then included in the decision tree model to predict RRT use. The decision tree model identified creatinine as the most important discriminating factor, followed by RAP only in patients with creatinine >2.7 mg/dL (Figure 1). Regarding model calibration, R2 was 0.90 with intercept -0.02 and slope of 0.94. AUC-ROC was 0.81 (0.62-0.97; 95% CI, p < 0.01), sensitivity = 58.3% and specificity = 100%.

**Figure 2:**
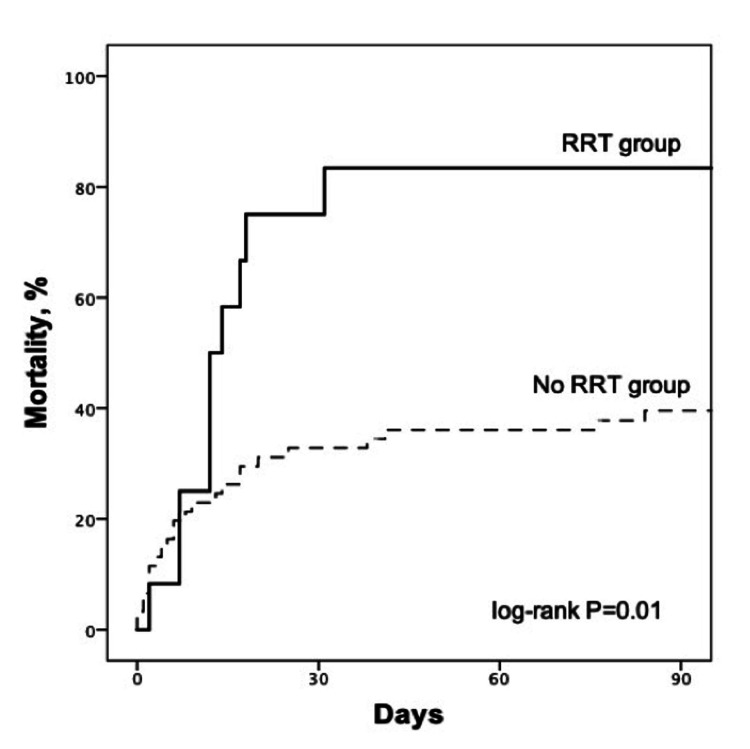
Ninety-day mortality for all patients, divided in two groups according to RRT use or not. RRT, renal replacement therapy

ICU and in-hospital mortality of patients who used RRT were significantly higher than in patients who did not use RRT (75.0% vs. 29.5% and 75.0% vs. 34.4%, respectively, p < 0.01 for both comparisons). The 90-days mortality was significantly higher in the RRT group when compared to no RRT group (83.3% vs. 42.6%, p = 0.01). Kaplan-Meier curves categorized by RRT vs. no RRT are shown in Figure 2 (log-rank p = 0.01).

## Discussion

Intravenous diuretics are first-line treatment to obtain negative fluid balance and then reduce right ventricular preload and optimize blood volume in ADPH. Diuretic treatment alone may not be enough to treat fluid overload in ADPH due to diuretic resistance, worsening kidney function or organ congestion, all of which contribute to the development of the cardiorenal syndrome [4-5, 16]. In the present study, all patients were under IV diuretics at ICU admission, dose was individualized by the ICU team based on degree of fluid overload and baseline kidney function. 

Renal replacement therapy is recognized as a reasonable treatment for patients with acute left heart failure (ALHF) with refractory fluid overload, cardiorenal syndrome or diuretic resistance [17-18]. In contrast, RRT role in ADPH is still unclear, there is no prospective data or clinical trials in ADPH. Concerns do exist, especially regarding the effect of RRT on short- and long-term outcomes.

Sztrymf et al. demonstrated that in ADPH the most common indications for RRT were oligoanuria, uremia, and refractory fluid overload [6]. In the present study the main reason for RRT initiation was diuretics refractory fluid overload. In the setting of refractory fluid overload, we challenged patients with IV diuretics for a median of two days before considering RRT. Continuous venovenous hemodiafiltration was the most common method in our study due to the presence of hemodynamic instability. The optimal timing for initiation of RRT and which modality to choose as the initial therapy is still under debate [19].

The AKI can lead to dysregulation of the renin-angiotensin-aldosterone system, increased sympathetic nervous system activity, and impaired sodium excretion, leading to fluid overload, which can be represented by elevated RAP [20]. Elevated RAP is a poor prognostic sign. It reflects a constellation of anatomical changes to deal with the chronically increased afterload and increased right ventricular filling pressures due to severe right ventricular dysfunction [21].

Our decision tree suggests a significant interaction between RAP and AKI as predictors of RRT use in ADPH. We developed and internally validated a decision tree model which highlights a 100% RRT use in ADPH patients with creatinine >2.7 mg/dL and RAP > 9 cmH20. RAP was useful for refinement of the model, suggesting that hypervolemic patients are at increased risk to go to RRT when they are admitted with severe AKI.

In patients with severe AKI and refractory hypervolemia, RRT represents a cornerstone of treatment [22]. Early RRT for ultrafiltration seems an appealing strategy to rapidly remove fluids and possibly improve outcomes in ADPH [4-5]. However, the current data show that RRT in unstable PAH/CTEPH patients may be dangerous and is associated with hypotension and severe adverse events including deaths during RRT sessions [6]. Our data did not show deaths during RRT but we had frequent episode of transient hypotension and malfunctioning of lines and dialysis circuit. 

For patients with admission creatinine <1.24 mg/dL there was no need for RRT in our sample. Those patients were challenged with IV diuretics and were able to keep negative fluid balance. On the other hand, for the group with admission creatinine between 1.24 and 2.7 mg/dL our model could not predict use of RRT even with RAP values included in the model. Observational data suggest that patients with mild AKI will respond to furosemide better than patients with severe AKI [23]. We believe that this intermediate group with mild/moderate AKI still benefits from IV diuretic challenge but RRT decision should be individualized. Therefore, RRT should be considered as an acceptable treatment in ADPH patients with life-threatening complications of severe AKI and refractory hypervolemia although the actual benefit in outcomes are still unclear [24-25].

In stable patients with PAH, AKI and chronic kidney disease are associated with a worse hemodynamic profile and are predictors of mortality [21, 26]. In the landmark study about RRT in ADPH, Sztrymf et al. showed that elevated creatinine at admission was associated with ICU mortality. In that study, when RRT was used, ICU mortality was 46.7% and 90-days mortality was 73.3% [6]. Haddad et al. showed that AKI was common and strongly associated with 30-day mortality after acute right-side heart failure hospitalization (OR 5.3, 95% CI 2.2-13.2) [7].

In our cohort, most patients who required RRT had CTEPH. The relationship between non-surgical CTEPH and AKI and RRT is poorly studied. Patients with CTEPH usually are older that patients with PAH and CKD are associated with CTEPH in some cohorts [27]. Further studies are needed in this area to clarify the relationship between ADPH, RRT, and CTEPH.

Intensive care unit and in-hospital mortality of patients who used RRT were significantly higher than in patients who did not use RRT and it was comparable to previous studies [6, 13]. Need for RRT itself is a predictor of poor outcomes in critical care population [28]. The best timing to initiate RRT, who is the best candidate, the best modality, and how to avoid complications in ADPH are still under debate.

The present study has a retrospective observational nature and has some limitations. First, the small number of events limiting the number of variables that could have entered in the multivariable model can lead to presence of confounders and selection bias. Second, all patients were receiving IV loop diuretic regimen so we cannot exclude that it could be related to AKI in some cases. Third, central venous pressure measurements were not available in most patients during admissions so we could not use it as a surrogate for hypervolemia.

This study has several strengths. First, this is a unique study about RRT in ADPH, the second study in this population. Second, we included a homogenous population of pre-capillary PH. Third, due to the rarity of this condition a randomized trial would be unfeasible in this population; this observational study offer some initial insights in predicting RRT in ADPH. 

## Conclusions

Acute kidney injury is a frequent complication in patients admitted with ADPH, although RRT is rarely needed. High ICU, in-hospital, and 90-days mortality were observed in patients with ADPH who used RRT. The decision tree model based on creatinine upon ICU admission and RAP, a surrogate of hypervolemia, can identify patients at risk for RRT on ICU admission, however, RRT role in ADPH is still unclear.
